# Off the back burner: diverse and gender-inclusive decision-making for COVID-19 response and recovery

**DOI:** 10.1136/bmjgh-2020-002595

**Published:** 2020-05-07

**Authors:** Sulzhan Bali, Roopa Dhatt, Arush Lal, Amina Jama, Kim Van Daalen, Devi Sridhar, Women in Global Health, and Gender and COVID-19 Working Group, Clare Wenham

**Affiliations:** 1 Women in Global Health, Washington, DC, USA; 2 Somali Institute for Development Research and Analysis (SIDRA), Nairobi, Kenya; 3 Women in Global Health, Mogadishu, Somalia; 4 Department of Public Health and Primary Care, University of Cambridge, Cambridge, Cambridgeshire, UK; 5 Medical School, The University of Edinburgh, Edinburgh, UK

**Keywords:** epidemiology, health policy, infections, diseases, disorders, injuries

**Summary box**

- Epidemics are a gendered vulnerability, with their socioeconomic impact disproportionately high among women, even when, as it seems the case with COVID-19, mortality is higher among men.
- However, women are not only a vulnerable population, they can serve as agents of change whose contributions can improve epidemic response and recovery.
- In COVID-19 response and recovery, existing lack of diversity and gender representation in decision-making means perspectives of some of the most vulnerable communities are left out.
- The evidence and lessons from peace, disaster and business sectors suggest that lack of diversity and failing to leverage women’s expertise and talent in decision-making can limit an effective response.
- In addition to being ethical, diverse and gender inclusive decision-making will yield innovation and knowledge dividends, limit group-think and promote greater accountability for an adaptive response and resilient recovery to COVID-19.

Epidemics function as a gendered vulnerability, and yet gender remains an afterthought in health security and pandemic response, including to coronavirus disease 2019 (COVID-19).^1^ Emerging data indicate that COVID-19 mortality is greater among men, but past experiences suggest that the socioeconomic impact of epidemics tends to be far greater for women. As a result, it is essential to assess the intersectional and gendered vulnerabilities in health emergencies. In addition, given the gender-skewed landscape of power and decision-making in global health, it is also critical to outline women’s leadership and role in such contexts.

Women are users of health services, and they are agents of change in health, making critical contributions as parents, front-line responders, health promoters, influencers, researchers, scientists and decision-makers. In China’s COVID-19 response, female nurses and community health workers were the first line of defence against the outbreak.^1^ Despite their major role, an interplay of power and privilege often results in women—particularly women from minority ethnic groups—being under-represented in health and humanitarian leadership, even when women and such minorities are disproportionately affected. This is similarly the case for women from other under-represented groups such as indigenous people, in low and middle-income countries (LMIC) and sexual minorities or lesbian, gay, bisexual, transgender, queer and intersex (LGBTQI) individuals.

Regardless of constituting over 70% of the global health workforce, women hold only 25% of leadership roles in health.^2^ Further, women’s scientific expertise is often excluded in the public realm even though they are more engaged in science outreach.^3^ The imbalance in diversity and equity, at the intersection of gender and LMIC representation, is also mirrored in publications and editorial boards of major global health journals.^4 5^ A recent breakdown of WHO Expert Advisory Panels shows that only 34% of members were women and only 11% of members were from the African region, compared with 29% of members from the European region.^6^ These patterns of inequality in decision-making are then reflected in who sets the research priorities that inform policy, and who makes the policy decisions in COVID-19 response and recovery.

The lack of representation in agenda setting and decision-making matters because women face a triple burden in pandemics—high risk of exposure to nosocomial infections in their role as healthcare workers; lost opportunities due to school closures and increased unpaid family care; and heightened risk of ill health due to diverted resources and the cascading effects of pandemics.^1^ In the context of COVID-19, women and sexual minorities can also face a higher risk of Gender-Based Violence (GBV). Additionally, women constitute a majority of workers in the non-agricultural informal sector^7^—leaving them vulnerable to loss of livelihood and economic insecurity due to the disruptive ‘fearonomic effects’ of pandemics.^8^ The socioeconomic impact of disasters and crises is further exacerbated for women in minority and lower income groups.^9^ As mothers, women are more likely to be responsible for nearly 1.5 billion children who are out of school in times of widespread school disruptions—drastically impacting their ability to be financially independent.^10^

Epidemics further exacerbate other profound security, mental health and health risks in countries facing protracted conflicts with widespread gender inequality and general societal and economic disruption, due to weakened infrastructure and reduced access to health services. Lack of diversity and gender representation in decision-making at global, country and organisational levels means perspectives of some of the most vulnerable communities—including refugees and migrants, ethnic and sexual minorities—are often left out, limiting an effective response by failing to address the direct and indirect effects on women and girls and minorities, and failing to leverage their expertise and talent when it is needed most.

Gender-equal representation is more than just a moral imperative. When gender representation moves beyond pure symbolism, it leads to smarter, ethical and more effective decision-making—especially in crises. Evidence from peace and security studies shows that although peace processes led by women were 35% more likely to last, less than 13% of peace negotiators selected are women.^11^ Inclusion of women in decision-making advances stability and security, community trust and financial accountability, and focuses more on reducing inequities. Additionally, evidence from the business sector highlights the strong association of gender diversity with innovative and ethical decision-making, and with reduction in fraud and cognitive biases.^12–15^ Greater gender representation brings with it diverse perspectives and approaches to problem solving, resulting in faster and better quality decisions.^16 17^

For epidemic response to be effective and adaptive, perspectives of women, and LMICs and vulnerable communities are critical. Interventions that work in high-income countries may not work elsewhere. For example, border closures will have limited applicability in countries with porous borders, and top-down interventions may be less effective in countries where the community trust in governments is lower, particularly in areas where women have been mistreated by police and armed forces. Insights from the implementation of interventions in LMICs (for COVID-19 and previous disease outbreaks) and country-specific context that takes into perspectives of women, and vulnerable communities can greatly inform the COVID-19 response and recovery. A diverse decision-making body for health security and COVID-19 response, with expertise from LMICs, women, patient groups, indigenous people and the LGBTQI community, will limit group-think, boost problem solving, accountability and learning to unleash overlooked and innovative context-appropriate solutions to global challenges.

**Figure 1:**
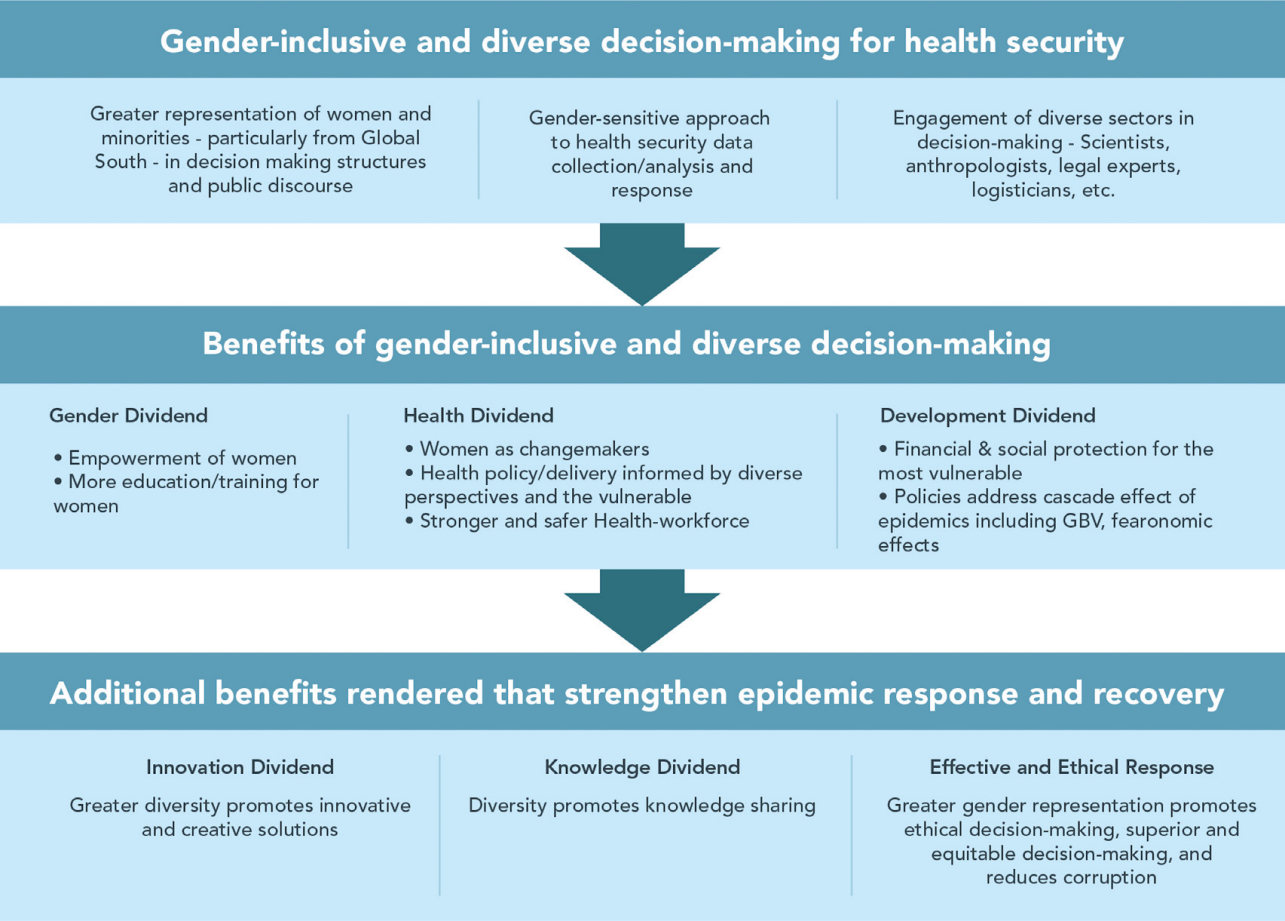
How greater gender representation and diversity in decision-making strengthens COVID-19 and other epidemic responses. GBV, Gender-Based Violence.

The same people who are engaged in response today will be the ones leading recovery and preparedness efforts tomorrow. Measures to promote gender equity, and representation in decision-making at all levels such as those proposed in the ‘COVID 50/50 Five Asks for Gender-Responsive Health Security’ will strengthen both global and local COVID-19 efforts and ensure greater engagement of those who are most affected in planning of response and recovery.^18^ In addition, mainstreaming gender in data collection and policy instruments such as the Joint External Evaluations and greater inclusion of women—particularly women from LMICs—in decision-making at the global level and in public discourse will ensure that we reap the innovation and knowledge dividends through smarter and creative decision-making for an effective COVID-19 response and a resilient post-COVID-19 future (see figure 1).
